# The Impact of the Organism on Its Descendants

**DOI:** 10.1155/2012/640612

**Published:** 2011-12-01

**Authors:** Patrick Bateson

**Affiliations:** Sub-Department of Animal Behaviour, University of Cambridge, High Street, Madingley, Cambridge CB23 8AA, UK

## Abstract

Historically, evolutionary biologists have taken the view that an understanding of development is irrelevant to theories of evolution. However, the integration of several disciplines in recent years suggests that this position is wrong. The capacity of the organism to adapt to challenges from the environment can set up conditions that affect the subsequent evolution of its descendants. Moreover, molecular events arising from epigenetic processes can be transmitted from one generation to the next and influence genetic mutation. This in turn can facilitate evolution in the conditions in which epigenetic change was first initiated.

## 1. Introduction

The view that knowledge of development was irrelevant to the understanding of evolution was forcefully set out by the advocates of the Modern Synthesis [1]. They brought the mechanism for the evolution of adaptations originally proposed by Darwin and Wallace together with Mendelian and population genetics. Maynard Smith [2] suggested that the widespread acceptance of Weismann's [3] doctrine of the separation of the germline from the soma was crucial to this line of thought even though it did not apply to plants. Such acceptance led to the view that genetics and hence evolution could be understood without understanding development. These views were, until recently, dominant. Briefly put, genes influence the characteristics of the individual; if individuals differ because of differences in their genes, some may be better able to survive and reproduce than others and, as a consequence, their genes are perpetuated.

The extreme alternative to the modern synthesis is a caricature of Lamarck's views about biological evolution and inheritance. If a blacksmith develops strong arms as a result of his work, it was argued, his children will have stronger arms than would have been the case if their father had been an office worker. This view has been ridiculed by essentially all contemporary biologists. Nevertheless, as so often happens in polarised debates, the excluded middle ground concerning the evolutionary significance of development and plasticity has turned out to be much more interesting and potentially productive than either of the extreme alternatives. This view was developed at length by West-Eberhard [4] who argued that developmental plasticity was crucial in biological evolution. These same ideas are well expressed in Gilbert and Epel's [5] book and developed further in the book edited by Pigliucci and Müller [6].

Bateson and Gluckman [7] have argued that developmental plasticity is an umbrella term for multiple unrelated mechanisms. The term includes accommodation to the disruptions of normal development caused by mutation, poisons, or accident. Much plasticity is in response to environmental cues, and advantages in terms of survival and reproductive success are likely to arise from the use of such mechanisms [7]. An organism that has been deprived of certain resources necessary for development may be equipped with mechanisms that lead it to sacrifice some of its future reproductive success in order to survive. Plasticity includes preparing individuals for the environments they are likely to encounter in the future on the basis of maternal cues; the course of an individual's development may be radically different depending on the nature of these cues. Plasticity may also involve one of the many different forms of learning, ranging from habituation through associative learning to the most complex forms of cognition.

I will not deal extensively with all the various ways in which an individual can affect the evolution of its descendants since I have discussed them recently elsewhere [8]. To summarise my position on this topic, I believe that the organism's mobility, its choices, its construction of a niche for itself, its capacity for behavioral innovation, and its adaptability have all played important roles in biological evolution. All these activities should be contrasted with the essentially passive role often attributed to the organism by many evolutionary biologists. Modern understanding of an individual's development goes well beyond accepting that interactions between the organism and its environment are crucial. The conditional character of an individual's development emphasises the need to understand the processes of development that underlie these interactions.

## 2. The Importance of Epigenetics

Epigenetics is a term that has had multiple meanings since it was first coined by Waddington [9]. He used the term, in the absence of molecular understanding, to describe processes by which the inherited genotype could be influenced during development to produce a range of phenotypes. He distinguished “epigenetics” from the eighteenth-century term “epigenesis,” which had been used to oppose the preformationist notion that all the characteristics of the adult were already present in the embryo.

More recently, the term epigenetics has been used for the molecular processes by which traits, specified by a given profile of gene expression, can persist across mitotic cell division without involving changes in the nucleotide sequence of the DNA. (Nowadays this usage is also taken to include transgenerational inheritance as discussed below.) In this more restricted sense, epigenetic processes are those that result in the silencing or activation of gene expression through such modification of the roles of DNA or its associated RNA and protein. The term has, therefore, come to describe those molecular mechanisms through which both dynamic and stable changes in gene expression are achieved, and ultimately how variations in extracellular input and experience by the whole organism of its environment can modify regulation of DNA expression [10]. This area of research is one of the most rapidly expanding components of molecular biology. It should be noted, however, that some authors [11], myself among them, continue to use Waddington's broader definition of epigenetics to describe all the developmental processes that bear on the character of the organism. In all these usages, epigenetics usually refers to what happens within an individual developing organism.

Variation in the context-specific expression of genes, rather than in the sequence of genes, is critical in shaping individual differences in phenotype. This is not to say that differences in the sequences of particular genes between individuals do not contribute to phenotypic differences, but rather that individuals carrying identical genotypes can diverge in phenotype if they experience separate environmental experiences that differentially and permanently alter gene expression.

The molecular processes involved in phenotypic development were initially worked out for the regulation of cellular differentiation and proliferation [5]. All cells within the body contain the same genetic sequence information, yet each lineage has undergone specialisations to become a skin cell, hair cell, heart cell, and so forth. These phenotypic differences are inherited from mother cells to daughter cells. The process of differentiation involves the expression of particular genes for each cell type in response to cues from neighbouring cells and the extracellular environment and the suppression of others. Genes that have been silenced at an earlier stage remain silent after each cell division. Such gene silencing provides each cell lineage with its characteristic pattern of gene expression. Since these epigenetic marks are faithfully duplicated across mitosis, stable cell differentiation results. These mechanisms are likely to play many other roles in development, including the mediation of many aspects of developmental plasticity.

A growing body of evidence suggests that phenotypic traits established in one generation by epigenetic mechanisms may be passed directly or indirectly through meiosis to the next, involving a variety of different processes, some involving microRNAs and some involving maternal behaviour [12]. In itself, this evidence does not relate to the thinking about biological evolution because the trans-generational epigenetic effects could wash out if the conditions that triggered them in the first place did not persist. The crucial question is to ask how epigenetic changes that are not stable could lead to genetic changes. I suggest that the answer subdivides into two likely routes for an evolutionary change in the genome.

## 3. Epigenetics as a Driver of Evolution

The first account of how a phenotypic change induced by a change in the environment could lead to a change in the inherited genome was provided by Spalding [13]. His paper is also historically important because it provides the first clear account of behavioural imprinting with which Lorenz [14] is typically associated.

Spalding's driver of evolution comprised a sequence of learning followed by differential survival of those individuals that expressed the phenotype more efficiently without learning. The same idea was advanced once again by Baldwin [15], Lloyd Morgan [16], and Osborn [17], all publishing in the same year. Seemingly, their ideas were proposed independently of Spalding and, indeed, of each other, although they may have unconsciously assimilated what Spalding wrote 23 years earlier in what was a widely read journal, *Macmillan's Magazine*, the predecessor of today's *Nature.*

Regardless of how they derived their ideas, the evolutionary mechanism proposed by Spalding and then Baldwin, Lloyd Morgan, and Osborn was known at the time as “organic selection” and is now frequently termed the “Baldwin effect,” largely because of Baldwin's influential book [18]. Baldwin was not always consistent in how he thought about the process, and, as a result, modern usage is confused [19]. By contrast, Lloyd Morgan's account of the process was particularly clear. He suggested that if a group of organisms respond adaptively to a change in environmental conditions, the modification will recur generation after generation in the changed conditions, but the modification will not be inherited. However, any variation in the ease of expression of the modified character which is due to genetic differences is liable to act in favour of those individuals that express the character most readily. As a consequence, an inherited disposition to express the modifications in question will tend to evolve. The longer the evolutionary process continues, the more marked will be such a disposition. Plastic modification within individuals might lead the process, and a change in genes that influence the character would follow; one paves the way for the other.

Given Spalding's precedence and the simultaneous appearance in 1896 of the ideas about “organic selection,” it seems inappropriate to term the evolutionary process the “Baldwin effect,” particularly since it has not been used consistently [19]. Calling the proposed process the “Spalding effect” is not descriptive of what initiates the hypothetical evolutionary process. West-Eberhard's [4] term “genetic accommodation” is more general but makes no inference about the inducing pathway; it would therefore be more appropriate to employ a term that captures the adaptability of the organism in the evolutionary process, and, to this end, I have suggested the term “adaptability driver” [20].

While the focus of Baldwin, as a psychologist, was largely on behaviour as the form of phenotypic response that was, in some way, incorporated over time into the genome, the model also allows for other forms of adaptive or plastic response to be thus incorporated. All that is required is that the adaptability in some way confers advantage in the novel environment, be it a physiological response such as coping with high altitudes by enhancing the oxygen-carrying capacity of the blood, or a change in coloration that improves concealment against predators, or a change in tail morphology in the tadpole that reduces the risk of predation. Over time, genetic accommodation can fix the alteration in the lineage. As the evolutionary change progressed, the population would consist of individuals with the same phenotype but which developed in different ways, some by their capacity to respond adaptively to environmental challenges and some by spontaneously expressing part or all of the phenotype without employing plastic mechanisms.

A clear case of adaptability driving evolutionary change may be that of the house finch (*Carpodacus mexicanus*). In the middle of the twentieth century, the finch was introduced to eastern regions of the USA far from where it was originally found on the west coast. It was able to adapt to the new and extremely different climate and spread up into Canada. The finch also extended its western range north into Montana, where it has been extensively studied. After a period involving great deal of plasticity, the house finch populations spontaneously expressed the physiological characteristics that best fitted them to their new habitats without the need for developmental plasticity [21].

The question remains: under what circumstances will fixation of a previously plastic phenotype occur? The chances that all the mutations or genetic reorganisations necessary to give rise to genetic fixation would arise at the same time are small. To take a behavioural example, if a phenotype expressed spontaneously without being learned is not as good as the learned one (in the sense that it is not acquired more quickly or at less cost), then nothing will happen and fixation will not occur. If the spontaneously expressed phenotype is better than the learned one, evolutionary change towards fixation is possible. If learning involves several subprocesses, as well as many opportunities for “chaining” (the discriminative stimulus for one action becoming the secondary reinforcer that can strengthen another action), then the chances against a spontaneously expressed equivalent appearing in one step are small. However, with learning processes available to fill in the gaps of a sequence, every small evolved step that cuts out the need for a plastic component while providing a simultaneous increase in efficiency is an improvement.

Simpson [22] thought that the proposed evolutionary change would lead to a generalised loss of the ability to learn. Quite simply, it would not. Learning in complex organisms consists of a series of subprocesses [23]. A particular activity can evolve to a point where it is expressed spontaneously without involving plastic process without any more generalised loss of plasticity. It remains to be seen whether similar arguments can be applied cogently to other forms of phenotypic change, where the plastic response has been physiological or anatomical. When a plastic change involves a system that does not have parallel architecture with built-in redundancies, then the cost of losing it could outweigh the benefits of increasing the efficiency of response to an environmental challenge.

## 4. Epigenetics as a Driver of Mutation

A wide variety of changes in endocrine regulation following developmental stresses are mediated by epigenetic mechanisms in experimental animals [7]. Induced epigenetic changes have also been described in naturally occurring plants [6]. The evidence for transmission across generations in both animals and plants continues to grow [12]. Epigenetic inheritance over at least eight generations has been reported in the plant *Arabidopsis* [24]. One research programme on mice examined individuals possessing a *Kit* paramutation (a heritable, meiotically stable epigenetic modification resulting from an interaction between alleles in a heterozygous parent) that results in a white-spotted phenotype. Injection of RNA from sperm of heterozygote mice into wild-type embryos led to the white-spotted phenotype in the offspring, which was in turn transmitted to their progeny [25]. In another study, mouse embryos were injected with a microRNA that targets an important regulator of cardiac growth. In adulthood, these mice developed hypertrophy of the cardiac muscle, which was passed on to descendants through at least three generations without loss of effect [26]. Furthermore, the microRNA was detected in the sperm of at least the first two generations, thus implicating sperm RNA as the likely means by which the pathology is inherited. The possible involvement of sperm is also supported by observations that transgenerational genetic effects on body weight and appetite can be passed epigenetically through the mouse paternal germline for at least two generations [27].

Male rats were exposed *in utero* to the endocrine disruptor vinclozolin during the sensitive period for testis sex differentiation and morphogenesis. Lowered spermatogenic capacity and several adult-onset diseases were observed over four successive generations; these were accompanied by altered DNA methylation patterns in the germline [28, 29]. Further analysis of these male offspring revealed that vinclozolin decreased methylation levels of two paternally imprinted genes and increased that of three maternally imprinted genes [30]. The work on *Arabidopsis* and mice suggests that micro-RNA may provide the means for transmission of methylation marks from one generation to the next [25, 31].

In most experimental studies, the environmental stimulus producing an epigenetic change is only applied in one generation. This might be enough since work on yeast suggests that an environmental challenge can permanently alter regulation of genes [32]. In natural conditions, the environmental cues that induce epigenetic change may be recurrent and repeat what has happened in previous generations. This recurring effect might stabilise the phenotype until genetic accommodation and fixation have occurred. Alternatively, DNA silencing may be stable as, for example, in *Linaria* [33] in which the epigenetically induced phenotype does not change from one generation to the next.

A central question in considering evolutionary change driven by the environment is whether the transmitted epigenetic markers could facilitate genomic change [34]. The answer is that, in principle, they could if (a) they were transmitted from one generation to the next, (b) they increased the fitness of the individual carrying the markers, and (c) genomic reorganisation enabled some individuals to develop the same phenotype at lower cost. Epigenetic inheritance would serve to protect the well-adapted phenotypes within the population until spontaneous fixation occurred. That much is exactly the same as has been proposed for the operation of the adaptability driver. However, another process could be at work.

DNA sequences where epigenetic modifications have occurred may be more likely to mutate than other sites. The consequent mutations could then give rise to a range of phenotypes on which Darwinian evolution could act. If epigenetic change could affect and bias mutation rates, such nonrandom mutation would facilitate fixation.

Methylated CpGs are mutational hotspots due to the established propensity of methylated cytosine to undergo spontaneous chemical conversion to thymine and methylated guanine to convert to uracil [35]. As these are functional nucleotides, they are not recognised as damaged DNA and excised or corrected by DNA repair mechanisms. Thus, the mutation becomes incorporated in subsequent DNA replications. DNA mapping shows fewer CpG sequences in the DNA than expected [36], and CpG hypermutability has led to a decrease in frequency of amino acids coded by CpG dinucleotides in some organisms. Indeed, comparison of the human and chimpanzee genomes has shown that 14% of the single amino acid changes are due to the biased instability of CpG sequences, which can be subject to methylation and thence to mutations [37]. The methylation of CpGs is a major contributing factor to mutation in *RB1*, a gene in which allelic inactivation leads to the developmental tumour, retinoblastoma [38].

Further evidence in support of the hypothesis that epigenetic change can lead to mutation is found in the analysis of neutrally evolving strands of primate DNA. The evidence indicates that the phylogenetically “younger” sequences have a higher CpG content than the “older” sequences, due to the reduced opportunity for spontaneous mutation. Intriguingly, the CpG content is strongly correlated with a higher rate of neutral mutation at non-CpG sites [39, 40], which suggests that CpGs play a role in influencing the mutation rate of DNA not containing CpG, perhaps by influencing the chromatin conformation surrounding the CpG and making it more accessible to other modifying processes. Furthermore, CpG content also appears to influence the *type of mutation* that occurs, with a higher ratio of transition-to-transversion mutations observed in parallel with the non-CpG mutation rate [40].

## 5. Implications for Evolutionary Novelty and Speciation

Major transitions in evolution have been explained in terms of changes in genetic organisation [41], and such changes have been offered as an explanation for the explosion of variety seen in the Cambrian era [42, 43]. Transitions in the rate of evolution can involve the remodelling of existing structure by changes in which part of a regulatory gene is expressed and when in development it is expressed [44]. Some of this might involve epigenetic mechanisms. The occasional appearance of mutations and the reorganisation of the genome permit evolutionary change that would not have previously been possible. Gene duplication provides a substrate on which new features can be added while sustaining existing phenotypic characteristics.

Many years ago, Riedl [45] argued that the structure of an organism made certain types of evolutionary change more probable than others. Dawkins [46] noted that when he introduced the possibility for segmentation within his computer-generated biomorphs, he was able to obtain variation that he had not found without such a developmental capability. This general point about the role of development in evolution has enormously important implications for the understanding of evolutionary processes, and the issue of evolvability continues to excite considerable debate [47]. What makes one lineage evolve more rapidly than another has already opened up the new science of “evo-devo” [42, 43]. The role of epigenetic change in driving novel mutational substrates, as discussed above, provides further opportunities for phenotypically driven evolutionary change. This point is discussed further in the final chapter of the book edited by Gissis and Jablonka [12].

More speciation occurs within a clade when polyphenism occurs within that clade [48]. This suggests that the presence of developmentally induced polyphenism favours adaptive radiation, providing a range of niche-defined phenotypes on which Darwinian evolution can act after fixation of the epigenetically mediated difference. Such a set of processes is likely, for example, to have occurred in a violet, *Viola cazorlensis* [49]. In this case, epigenetic differentiation of populations was correlated with adaptive genetic divergence.

King [50] suggested that speciation often involves a change in chromosome number. The number is known to be under genetic control. Closely related species can be strikingly different. In horses, for example, the chromosome number ranges from 32 in *Equus zebra hartmannae* and 46 in *Equus grevyi* to 62 in *Equus assinus* and 66 in *Equus przewalski*; all but two of the horse hybrids are sterile. Similar variations in chromosomal number have been found in other mammals and strikingly in Alpine populations of house mice [51]. Humans and chimpanzees have different chromosomal numbers; chromosome 2 of the human is a fusion of two ancestral chromosomes, denoted 2A and 2B in the chimpanzee [52]. How could these differences between closely related species arise in evolution without involving the problems encountered by a solitary “hopeful monster” [53]? A hypothetical example illustrates one way.

Suppose that a herd of zebras wanders away from its usual habitat and enters an area where many of the plants available to the zebras as food contain toxins which they had not previously experienced. These toxins exert a developmental impact on the fetuses carried by the mares, and they form characteristics that are novel. When born, the zebra foals cope through phenotypic accommodation, but this nevertheless occurs at significant cost. In time, and in some individuals, these costs are minimised by genetic changes—perhaps biased by epigenetic change—and the type of evolutionary mechanism proposed by Darwin and Wallace operates to the advantage of these individuals and their offspring. Over time, the reorganisation required by such changes cascades and more and more genetic changes appear as the evolutionary adaptation processes create new order in the regulation of the zebra's development. The final step in this conjecture is that the genomic reorganisation impacts on chromosome number since the number is under genetic control. If this happens, then a reproductive barrier would be established between the new zebra population and the one from which it originated.

My general point is that an individual's adaptability allows a lineage to occupy a new place which can then lead to descendants entering many unexploited niches within that new habitat. The Galapagos finches are a clear example of how, in a relatively short space of time, birds arriving from the mainland were able to radiate out into many different habitats [54]. Tebbich et al. [55] discuss how the finches' capacity to respond to environmental challenges, for which they provide some evidence, could have played an important role in this process. None of this challenges the evolutionary mechanism postulated by Charles Darwin and Alfred Russel Wallace. The evolutionary process requires variation, differential survival and reproductive success, and inheritance. Three questions for the modern study of epigenetics arise from this formulation. First, what generates variation in the first place? Second, what leads to differential survival and reproductive success? Third, what factors enable an individual's characteristics to be replicated in subsequent generations? In answering all of these questions, an understanding of development is crucial.

## 6. Conclusions

One of the near-universal aspects of biology is that genetically identical individuals are able to develop in such strikingly different ways. Phenotypic variation can be triggered during development in a variety of ways, some mediated through the parent's phenotype. Sometimes phenotypic variation arises because the environment triggers a developmental response that is appropriate to those ecological conditions [56, 57]. Sometimes the organism “makes the best of a bad job” in suboptimal conditions. Sometimes the buffering processes of development may not cope with what has been thrown at the organism, and a bizarre phenotype is generated. Whatever the adaptedness of the phenotype, each of these effects demonstrate how a given genotype will express itself differently in different environmental conditions.

The decoupling of development from evolutionary biology could not hold sway forever. Whole organisms survive and reproduce differentially, and the winners drag their genotypes with them [4]. The way they respond phenotypically during development may influence how their descendants' genotypes evolved and were fixed [7]. This is one of the important engines of evolution and is the reason why it is so important to understand how whole organisms behave and develop.

The characteristics of an organism may be such that they constrain the course of subsequent evolution or they may facilitate a particular form of evolutionary change. The theories of biological evolution have been reinvigorated by the convergence of different disciplines. The combination of developmental and behavioural biology, ecology, and evolutionary biology has shown how important the active roles of the organism are in the evolution of its descendants. The combination of molecular biology, palaeontology, and evolutionary biology has shown how important an understanding of developmental biology is in explaining the constraints on variability and the direction of evolutionary change.
